# Evidence for cognitive resource imbalance in adolescents with narcolepsy

**DOI:** 10.1007/s11682-017-9706-y

**Published:** 2017-03-20

**Authors:** Suzanne T. Witt, Natasha Morales Drissi, Sofie Tapper, Anna Wretman, Attila Szakács, Tove Hallböök, Anne-Marie Landtblom, Thomas Karlsson, Peter Lundberg, Maria Engström

**Affiliations:** 1 Center for Medical Image Science and Visualization (CMIV), Linköping University, Linköpings universitet/US, SE-581 85 Linköping, SE Sweden; 2 Department of Medical and Health Sciences (IMH), Linköping University, Linköping, Sweden; 3 Radiation Physics, Department of Medical and Health Sciences, Linköping University, Linköping, Sweden; 4 Linnaeus Center HEAD, Linköping University, Linköping, Sweden; 5 Department of Pediatrics, Institute of Clinical Sciences, Sahlgrenska Academy, University of Gothenburg, Gothenburg, Sweden; 6 Department of Neurology, Department of Clinical and Experimental Medicine, Linköping University, Linköping, Sweden; 7 Department of Neuroscience and Neurology, Uppsala University, Uppsala, Sweden; 8 Department of Behavioral Sciences and Learning, Linköping University, Linköping, Sweden; 9 Radiology, Department of Medical and Health Sciences, Linköping University, Linköping, Sweden

**Keywords:** fMRI, Narcolepsy, Working memory, EEG, GABA, MRS

## Abstract

The study investigated brain activity changes during performance of a verbal working memory task in a population of adolescents with narcolepsy. Seventeen narcolepsy patients and twenty healthy controls performed a verbal working memory task during simultaneous fMRI and EEG acquisition. All subjects also underwent MRS to measure GABA and Glutamate concentrations in the medial prefrontal cortex. Activation levels in the default mode network and left middle frontal gyrus were examined to investigate whether narcolepsy is characterized by an imbalance in cognitive resources. Significantly increased deactivation within the default mode network during task performance was observed for the narcolepsy patients for both the encoding and recognition phases of the task. No evidence for task performance deficits or reduced activation within the left middle frontal gyrus was noted for the narcolepsy patients. Correlation analyses between the spectroscopy and fMRI data indicated that deactivation of the anterior aspect of the default mode in narcolepsy patients correlated more with increased concentrations of Glutamate and decreased concentrations of GABA. In contrast, deactivation in the default mode was correlated with increased concentrations of GABA and decreased concentrations of Glutamate in controls. The results suggested that narcolepsy is not characterized by a deficit in working memory but rather an imbalance of cognitive resources in favor of monitoring and maintaining attention over actual task performance. This points towards dysregulation within the sustained attention system being the origin behind self-reported cognitive difficulties in narcolepsy.

**Electronic supplementary material:**

The online version of this article (doi:10.1007/s11682-017-9706-y) contains supplementary material, which is available to authorized users.

## Introduction

Narcolepsy is a chronic sleep disorder characterized by excessive daytime sleepiness, cataplexy, frequent uncontrollable sleep attacks, and rapid eye movement sleep abnormalities such as sleep paralysis and hypnagogic or hypnopompic hallucinations. Patients with narcolepsy frequently report subjective complaints about working memory (Broughton et al. 1981), and results from studies attempting to objectively measure working memory deficits are mixed but mostly negative (Aguirre et al. 1985; Rogers and Rosenberg 1990; Smith et al. 1992), leading at least one study to conclude that there is little empiric evidence for a genuine memory deficit in narcolepsy (Hood and Bruck 1997). Instead, most studies report deficits in sustained attention (Fulda and Schulz 2001). The general pattern of cognitive dysfunction in narcolepsy is thought to be consistent with a misbalance of cognitive processing resources (Naumann et al. 2006) and may be related to changes in the hypocretin system (Peyron et al. 2000; Thannickal et al. 2000). Disruption of hypcretin-1 is associated with deficient regulation of vigilance and overall cortical activity (Rogers and Rosenberg 1990; Valley and Broughton 1981), so the loss of hypocretin-1 may lead to an instability of cortical activity disrupting the efficiency of cognitive control processes, such that the need to sustain attention at high levels over a long period of time adversely interferes with working memory performance (Naumann et al. 2006).

It is known that cognitive resources can be depleted during tasks that require the continuous allocation of attention (Helton and Russell 2011a, b; Warm et al. 2008). It has also been shown that the relative level of deactivation in the default mode network (DMN) can be indicative of current levels of attention (Anticevic et al. 2012; Weissman et al. 2006). Studies typically note decreased deactivation within the DMN during task performance as a marker for deficits in sustained attention (Bonnelle et al. 2011; Weissman et al. 2006). Under the proposed theory of cognitive resource and sustained attention dysfunctions in narcolepsy, one might expect to observe decreased deactivation of the DMN during cognitive task performance, reflecting increased distractibility and difficulty staying on task. However, given the current scarcity of functional neuroimaging studies investigating attention (or even cognitive) deficits in narcolepsy, the exact nature of any attention-related deficits commonly observed and reported in narcolepsy remains yet to be described.

γ-Amino-Butyric Acid (GABA) is the main inhibitory neurotransmitter in the human brain and is synthesized from Glutamate, the main excitatory neurotransmitter (Agarwal and Renshaw 2012; Platt 2007). A number of studies have shown a relationship between task-related BOLD responses and GABA concentrations, as measured using magnetic resonance spectroscopy (MRS) (Chen et al. 2005; Hu et al. 2013; Lauritzen et al. 2012; Muthukumaraswamy et al. 2009, 2012; Northoff et al. 2007). In humans, it has been demonstrated that resting-state GABA concentrations in the anterior cingulate cortex (ACC) were predictive of subsequently measured task-related negative BOLD responses in this same region (Northoff et al. 2007). Specifically, the authors noted that the higher the total GABA concentration, the stronger the negative BOLD response, suggesting that GABA may mediate negative BOLD responses, at least in the ACC (Northoff et al. 2007). It has also been reported that endogenous GABA levels correlated inversely with measured BOLD responses in the default mode network during performance of a working memory task (Hu et al. 2013). Very recently, it was demonstrated that plasma concentrations of a positive allosteric modulator of GABA were inversely correlated with BOLD responses in the ACC during performance of a working memory task, providing further evidence that GABA mechanisms mediate negative BOLD responses (Walter et al. 2016). As it has been previously demonstrated that young adults with narcolepsy exhibit increased GABA concentrations in the medial prefrontal cortex (Kim et al. 2008), examining the relationship between GABA concentration and task-related BOLD activity (both activations and deactivations) may further help elucidate the nature of any attention-related cognitive deficits in narcolepsy.

The purpose of this study was to investigate brain activity changes related to performance of a verbal working memory task using simultaneous functional magnetic resonance imaging (fMRI) and electroencephalography (EEG) and to correlate these to MRS-quantified levels of GABA and Glutamate in a population of adolescents with predominantly type 1 narcolepsy (narcolepsy with cataplexy). As a common self-report cognitive complaint in narcolepsy concerns working memory, we were interested in determining whether narcolepsy is characterized by a true working memory deficit, or if the current theory of a narcolepsy-related misbalance of cognitive resources is correct. Assuming that the proposed theory of finite cognitive resources is correct and that the neurocognitive deficits in narcoleptic patients stem from some form of dysfunction in the sustained attention system, we hypothesized that narcoleptic patients should exhibit decreased deactivation in the DMN during task performance compared with healthy participants during performance of a verbal working memory task. We additionally anticipated finding supporting evidence from both the EEG microstate and MRS analyses. For the EEG microstates, we anticipated finding evidence suggesting instability of the microstates, such as reduced mean duration or reduced global field power (Koenig et al. 2005). For the MRS data, we anticipated observing altered relationship between GABA concentrations and task-related BOLD deactivation.

## Methods

### Participants

A total of nineteen participants (aged 13–19.5 years) with a confirmed diagnosis of narcolepsy (Medicine 2005) were recruited from a population-based study in western Sweden, as well as pediatric clinics in Östergötland county. All patients met the criteria for type 1 narcolepsy, apart from one patient who did not have cataplexy and lacked measurement of CSF-hypocretin (subtyping was performed retrospectively according to the updated American Academy of Sleep Medicine guidelines (Medicine 2014)). An additional twenty-one healthy controls (aged 13.1–24.1 years) were recruited by advertisement to match the group-level age and gender distribution of the patients. All narcolepsy participants were allowed to take their prescribed medications prior to the exam. The healthy participants were confirmed to have no medical history of neurological diseases or mental illness through questionnaires and interviews prior to examination. To assess cognitive function, all participants were administered a series of previously published cognitive tests prior to MRI scanning (See Table 1. Tests were administered by AW and NMD, under the supervision of TK). Although a full clinical characterization of the narcolepsy participants has already been published (Szakacs et al. 2015), a summary of relevant demographic information together with cognitive testing results from a standard battery of test performed at the time of MRI scanning are provided (Table 1). All participants gave written informed assent (with written parental consent) or consent depending on their age according to the Declaration of Helsinki. Approval for the study was granted by the Regional Ethical Review Board in Linköping, Sweden (Dnr. 2013/99-31), and the study was conducted in accordance with the Helsinki Declaration.

**Table 1:** Summary of relevant demographic information and pre-scan cognitive assessment results. Values given as mean (standard deviation). Results from tests for between-group differences are shown in the final column

	Narcolepsy patients	Healthy controls	
Sample size	17	20	
Age	16.5 (1.9)	17.4 (2.6)	*t* = 1.13, *n.s.*
Gender	6 M/11 F	8 M/12 F	χ^2^ = 0.09, *n.s*.
Disease duration	3.7 (1.2)	-	
Medication status			
Methylphenidate (Modafinil, Medikinet, Ritalin, Concerta)	15/17	-	
Fluoxetine	7/17	-	
Depression symptoms†	7/17	-	
Pandemrix™ vaccine	16/17	-	
Word comprehension test^a^: # correct	10.4 (4.5)	12.6 (5.7)	*t* = 1.3, *n.s*.
Trail making test A^b^: Completion time	0:18 (0:07)	0:18 (0:03)	*t* = 0.11, *n.s.*
Trail making test B^b^: Completion time	1:03 (0:31)	0:58 (0:26)	*t* = 0.5, *n.s.*
Rey Osterreith complex figure Test^c,d^–1: Points	34.8 (1.4)	35.4 (1.2)	*t* = 1.4, *n.s.*
Rey Osterreith complex figure test^c,d^–2: Points	24.6 (8.2)	25.6 (4.8)	*t* = 0.4, *n.s.*
Digit span forward^e^	5.8 (0.8)	6.2 (1.1)	*t* = 1.2, *n.s.*
Listening span^f^	3.9 (0.7)	3.8 (0.6)	*t* = 0.96, *n.s.*
Listening span^f^: # correct	17.4 (4.3)	18.3 (4.17)	*t* = 0.6, *n.s.*

† Assessed at initial clinical visit

^a^Nilsson et al. (1997)

^b^Tombaugh (2004)

^c^Rey (1941)

^d^Osterrieth (1944)

^e^Wechsler (2008)

^f^Daneman and Carpenter (1980)

### Verbal working memory fMRI task

Working memory was assessed by means of a reading span task (Daneman and Carpenter 1980; Engstrom et al. 2013), in which participants studied sentences and decided whether each made sense (e.g., “The woman steered the car.”) or not (e.g., “The coffee cup steered the car.”) (Malm et al. 1998). In addition, participants were also asked to memorize the last word of each sentence. Following the presentation of a block of one, three, or five sentences, participants were presented four words, one at a time, and asked to respond via button press (Yes/No) if the word had been presented in the immediately preceding sentence block. Half of the words were targets, the remaining foils.

Eighteen sequences of sentences to encode and words to recognize were presented in pseudo-random order in an event-related fMRI design. Sentence blocks were presented at a rate of five seconds per sentence and included one, three, or five sentences. During the recognition phase, four words were presented for one second each, with a randomly jittered (Poisson distribution) inter-stimulus interval ranging from 1.5–6 s. Stimulus presentation and response recording were accomplished using Superlab 4.5 (Cedrus Inc., San Pedro, CA). Participants completed one run lasting approximately 12 min 45 s.

### MRI methods

All MR images were acquired on a 3 T Philips Ingenia (Philips Healthcare, Eindhoven, The Netherlands) located at the Center for Medical Image Science and Visualization (CMIV) at Linköping University, Sweden. FMRI data were acquired using a 32-channel head coil with a single-shot, gradient-echo EPI sequence (TR/TE = 2200/35 msec; SENSE factor = 2; FOV = 240 × 240 mm^2^; voxel =3 × 3 mm^2^; slice thickness (gap) = 3(0) mm; # slices =35; matrix =80; flip angle =77°; # volumes =347) that effectively covered the whole brain in 2.2 s.

MRS measurements were performed after the fMRI acquisitions using a MEGA-PRESS (Mescher et al. 1996; Mullins et al. 2014) sequence (TR/TE = 2000/68 msec, edited pulses ON at 1.90 ppm, edited pulses OFF at 7.46, water suppression MOIST, 40 dynamics) with the voxel (3x3x3 cm^3^) placed in the medial prefrontal cortex. Directly afterwards, a 2-dynamic unsuppressed water reference measurement was collected to obtain a reference of water in the tissue within the voxel, which was used for water scaling.

T1-weighted images were acquired and reviewed by a radiologist for all participants to ensure that they were otherwise free from any obvious pathologic abnormalities.

### fMRI stimulus delivery/response recording

Visual stimuli were presented using a projector system and displayed on a high resolution screen located behind the participant’s head. Participants viewed the screen using a mirror attached to the head coil. Corrective lenses were provided as needed. An MR-compatible fiber optic response device (Cedrus Inc., San Pedro, CA) was used to acquire behavioral responses.

### EEG recording

EEG was recorded (Vision Recorder, Brain Products GmbH, Gilching, Germany) inside the scanner using MR-compatible caps (Easycap GmbH, Herrsching, Germany) with 64 Ag/AgCl electrodes. The EEG montage was based on the 10–20 system positions. Electrocardiography (ECG) was measured from a separate electrode placed on the left side of a participant’s back. Data were recorded using a 5 kHz sampling rate, 32 mV input range, and 0.1–250 Hz bandpass filters. MR-compatible BrainAmp amplifiers (Brain Products GmbH) were placed in the back of the scanner bore and optical cables were passed through a wave guide in the wall of the scanner room to the recording equipment positioned outside of the scanner room. Due to technical problems with the EEG recording equipment, the data from three narcolepsy patients and three healthy controls were excluded from all EEG-related analyses.

### fMRI analysis

The EPI images were reconstructed on the scanner, and preprocessing was performed using SPM8 (The Wellcome Trust Centre for Neuroimaging, University College London, London, UK). All participants’ images were separately realigned using INRIAlign (Freire and Mangin 2001; Freire et al. 2002), and the translation and rotation correction parameters were individually examined to ensure that no participant had significant head motion larger than one voxel in any direction. This resulted in data from one healthy control being excluded from all further analyses. Spatial normalization into Montreal Neurologic Institute (MNI) space was initially performed on the mean functional image volume for each participant, and these normalization parameters were then applied to each respective functional image set. The normalized images were smoothed with an 8 mm FWHM Gaussian kernel.

The regressors from each participant’s fMRI model were derived by extracting the onset and duration timings for all task trials and modeled using a synthetic hemodynamic response function. The functional imaging data of each participant were modeled individually in SPM8 and included individual regressors for sentence blocks and word events (both incorrect and correct trials). The six motion-correction parameter estimates (*x*, *y*, and *z* displacements and pitch, role, and yaw rotations) were included as covariates of no interest to statistically control for signal change related to head motion. A high-pass filter (cut-off period =128 s) was incorporated into the model to remove low-frequency signals. All contrast images written by SPM8 represented brain activity relative to an ‘implicit baseline’ of unmodeled variance. To avoid including sentence blocks or word events that may have occurred during sleep, two consecutive missed events (e.g., no behavioral response) were determined to signify the beginning of sleep. These trials and all subsequent trials were removed on a per-participant basis; a return to wakefulness was indicated by two consecutive trials with correct responses. Significantly more events were removed for the narcolepsy patients than for the healthy controls, with an average of 20 ± 21 events removed for the narcolepsy patients and 3 ± 7 for the healthy controls (*t* = 3.4, *p* < 0.002), where the total number of events was 72. Trials with no behavioral responses that occurred in isolation (e.g., in between two trials with responses) were considered ‘missed’ and included in the GLM analyses.

Sentences were modeled as blocks of finite duration, while words were modeled as events of zero duration. A working memory loading factor, corresponding to the number of sentences presented during an encoding block (1, 3, or 5), was included as a parametric interaction term for both the sentence and word regressors. Contrasts corresponding to the encoding of sentences, the recognition of words, and the parametric effects of working memory load for sentences and words were specified for each participant. Overall group-level significance was assessed using 1-sample t-tests for each of the four contrasts of interest. Whole brain significance was assessed at *p* < 0.05, corrected for multiple comparisons via Family Wise Error (FWE).

### Region-of-interest analyses

To test our study hypothesis concerning altered activation of the DMN, as well as to test for the possibility of diminished activity in the left middle frontal gyrus (LMFG) given its key role in working memory, region-of-interest (ROI) analyses were used to test for significant between-group differences in the DMN and the LMFG. The DMN ROI used was that included in the CONN toolbox (Whitfield-Gabrieli and Nieto-Castanon 2012), based on published task negative results (Fox et al. 2005). The LMFG ROI was taken from the FSL Harvard-Oxford Atlas (Desikan et al. 2006; Frazier et al. 2005; Goldstein et al. 2007; Makris et al. 2006). For each ROI, the mean activation value was extracted on an individual subject basis for each of the four contrasts of interest using in-house code. Group difference was assessed across both ROIs and all four contrasts on interest simultaneously using a multivariate ANOVA controlling for age, gender, and number of missed responses. Significance of between-group effects were assessed at *p* < 0.05, Bonferroni corrected for multiple comparisons.

### Behavior analysis

To test for the effect of working memory load on task accuracy and reaction time, two separate repeated measures ANOVAs (controlling for age, gender, and number of missed trials) were performed. Correlation analyses between the behavioral and fMRI data were also performed to identify any relationships between task performance and relative neural activity in the hypothesized ROIs. Overall checks of the behavioral data led us to exclude two narcolepsy patients from all further analyses due to either missing more than 70% of trials and/or having an overall performance accuracy of less than 60%. This left a final study sample size of seventeen narcolepsy patients and twenty healthy controls. Overall significance of the ANOVA was assessed at an omnibus effect of *p* < 0.025, correcting for comparing across the two separate repeated measures ANOVAs.

### EEG analysis

EEG data were preprocessed using Analyzer 2.0 (Brain Products GmbH). First, data were segmented and corrected for MRI gradient and cardioballistic artifacts using standard template subtraction procedures (Pascual-Marqui et al. 1995). Independent component analysis was used to identify common artifact components (e.g., eye and shoulder/neck movement). Any remaining artifacts were rejected through visual inspection. Data were then downsampled to 125 Hz and bandpass filtered between 1 and 40 Hz (Pascual-Marqui et al. 1995). To investigate between-group differences in neural electrical activity during the recognition of words, microstate analysis was performed using a multi-step procedure referred to as ‘electrical neuroimaging’ (Michel et al. 2001, 2004), which comprises local and global measures of the electric field of the scalp and has been described in detail elsewhere (Michel et al. 2001, 2004; Pascual-Marqui et al. 1995). To capture the entire time of word events, post-stimulus epochs of 1000 msec were used. The electrical neuroimaging analysis was performed using the dedicated Cartool software by Denis Brunet (brainmapping.unige.ch/cartool). Separate, repeated-measures ANOVAs were performed to assess between-group differences in the mean duration, global field power, and average number of occurrences of across each microstate. Overall significance was assessed at *p* < 0.016, Bonferroni corrected for performing three separate tests. Post-hoc significance for comparing across the individual microstates was assessed at a Bonferroni corrected *p*-value for comparing across the number of unique, stable topographies.

### MRS analysis

The spectroscopy data were phase corrected according to Klose (1990) and frequency aligned based on the water residual. A difference spectrum was computed from the average ON and average OFF spectra. The difference spectrum was used as input to LCModel (Version 6.3-1E), which computed the GABA+ concentrations. As no macromolecular suppression was performed during the acquisition of the MRS data, the concentrations presented here are given in terms of GABA plus macromolecular contamination (GABA+) (Edden et al. 2012). The Glutamate concentrations were obtained solely by analyzing the OFF-dynamics. Two datasets (one narcolepsy patient and one healthy control) were discarded after visual interpretation due to excessive head motion during acquisition. Additionally, five data sets (four narcolepsy patients and one healthy control) were either not collected due to participant fatigue or excluded due to technical difficulties with the MEGA-editing.

In addition to examining between-group differences in the concentrations of GABA+ and Glutamate, Pearson’s correlations coefficients were calculated between the GABA+ and Glutamate concentrations and the working memory task-related activation extracted from 8 mm spherical ROIs placed at the stereotactic peaks of activation and deactivation in the medial prefrontal cortex (See Supplemental Table 1A-B) for the encoding of sentences and the recognition of words contrasts. A Fisher’s Z transformation (Fisher 1915, 1921) was used to test for between-group differences in the resulting correlation coefficients. Significance was assessed a *p* < 0.006, Bonferroni corrected for comparing across the eight separate between-group tests.

## Results

### fMRI activation

**Fig. 1:**
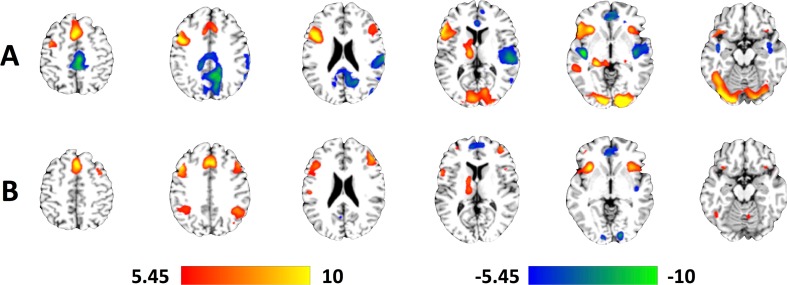
Representative axial slices of group-level fMRI activation during the working memory task across all subjects. **a** Encoding of sentences. **b** Recognition of words. All activation maps thresholded at *p* < 0.05, using Family Wise Error correction for comparing across all voxels in the brain. Color bars are scaled in terms of t-statistic. Slices were created using Mango (http://ric.uthscsa.edu/mango/; Jack L. Lancaster and Michael J. Martinez)

Overall group activation during the encoding of sentences and recognition of words are shown (Fig. 1a-b; peak stereotactic coordinates are provided in Supplemental Table 1A-B). As no clusters outside the visual cortices were observed for either the parametric effect of load during sentence encoding or the parametric effect of load during word recognition, group level activation maps are not displayed for these two contrasts. We refer readers, instead, to Supplemental Table 1C-D for a complete list of peak stereotactic coordinates these two contrasts. The general patterns of activation during both the encoding of sentences and the recognition of words were in line with what has been previously published and summarized in a recent quantitative meta-analysis (Rottschy et al. 2012).

**Fig. 2:**
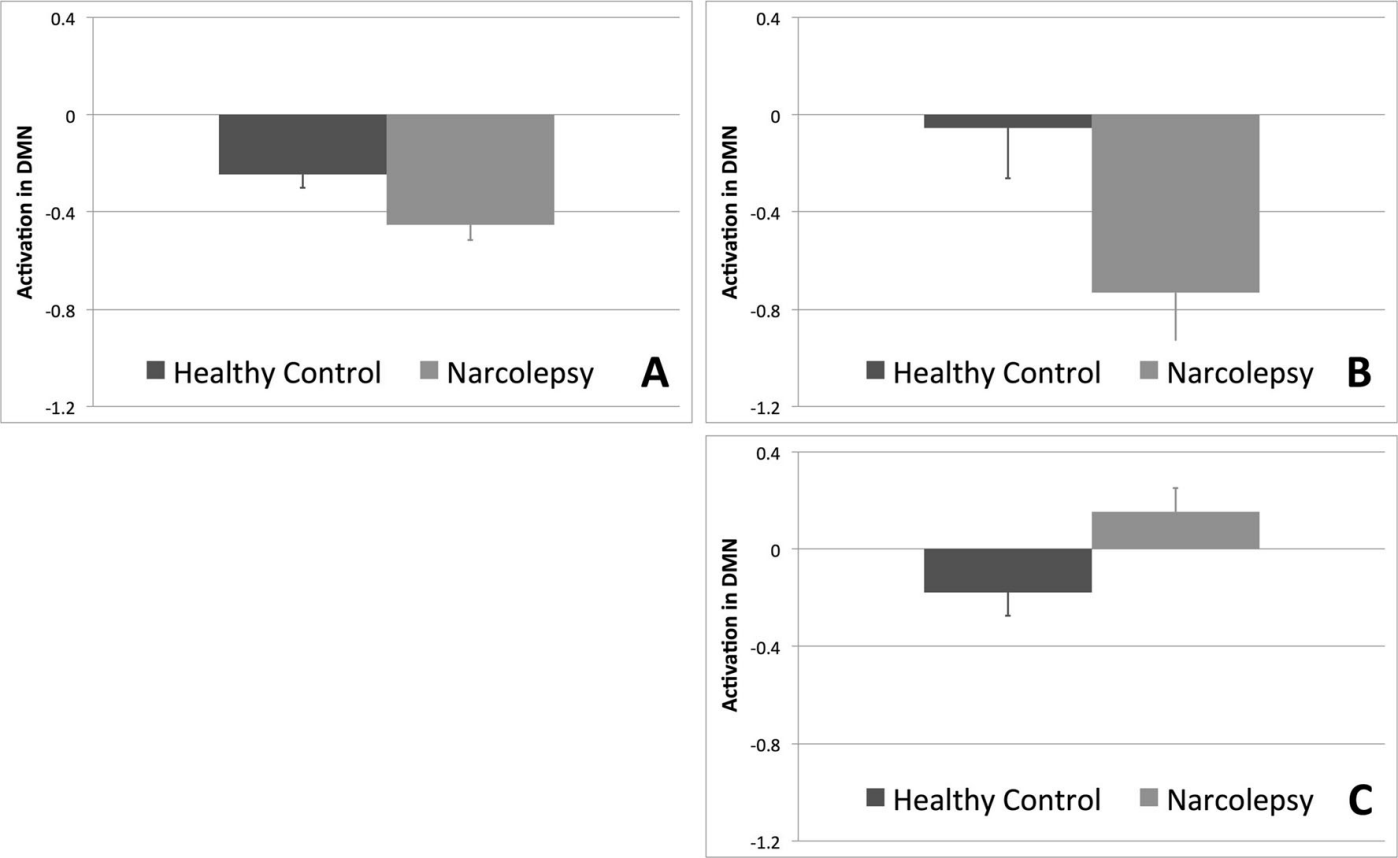
Results from between-group comparisons of activation within the default mode network (DMN) region-of-interest. **a** Encoding of sentences. **b** Recognition of words. C. Parametric effect of load during recognition of words. Activation displayed as estimated marginal means (corrected for age, gender, and number of missed trials) of beta values, with healthy controls shown in *dark gray* and narcolepsy patients in *light gray*. *Error bars* are given in terms of standard error

A significant omnibus multivariate effect was noted for study group (F_(8,26)_ = 2.425, *p* < 0.042) when examining group differences across both the LMFG and DMN ROIs for all four task contrasts of interest. Significant between-group effects were observed for the DMN ROI for the encoding of sentences (F_(1,33)_ = 6.228, *p* < 0.018), the recognition of words (F_(1,33)_ = 4.786, *p* < 0.036), and the parametric effect of load on the recognition of words (F_(1,33)_ = 5.898, *p* < 0.021) (Fig. 2a–c, Table 2). Planned comparisons revealed that the narcolepsy participants had significantly greater deactivation in the DMN during both the encoding of sentences and the recognition of words compared with the matched healthy controls. For the parametric effect of load on the recognition of words, the narcolepsy patients were observed to have positive activation within the DMN compared with deactivation in the healthy controls.

**Table 2:** Results from region-of-interest analysis for the working memory task. Significant planned between-group comparisons are given in values of estimated marginal means (corrected for age, gender, and number of missed trials) of beta values

	Narcolepsy patients	Healthy controls
Encoding of sentences	-0.46 ± 0.06	-0.245 ± 0.056
Recognition of words	-0.73 ± 0.2	-0.055 ± 0.21
Parametric effects of load, recognition of words	0.15 ± 0.10	-0.18 ± 0.09

**Fig. 3:**
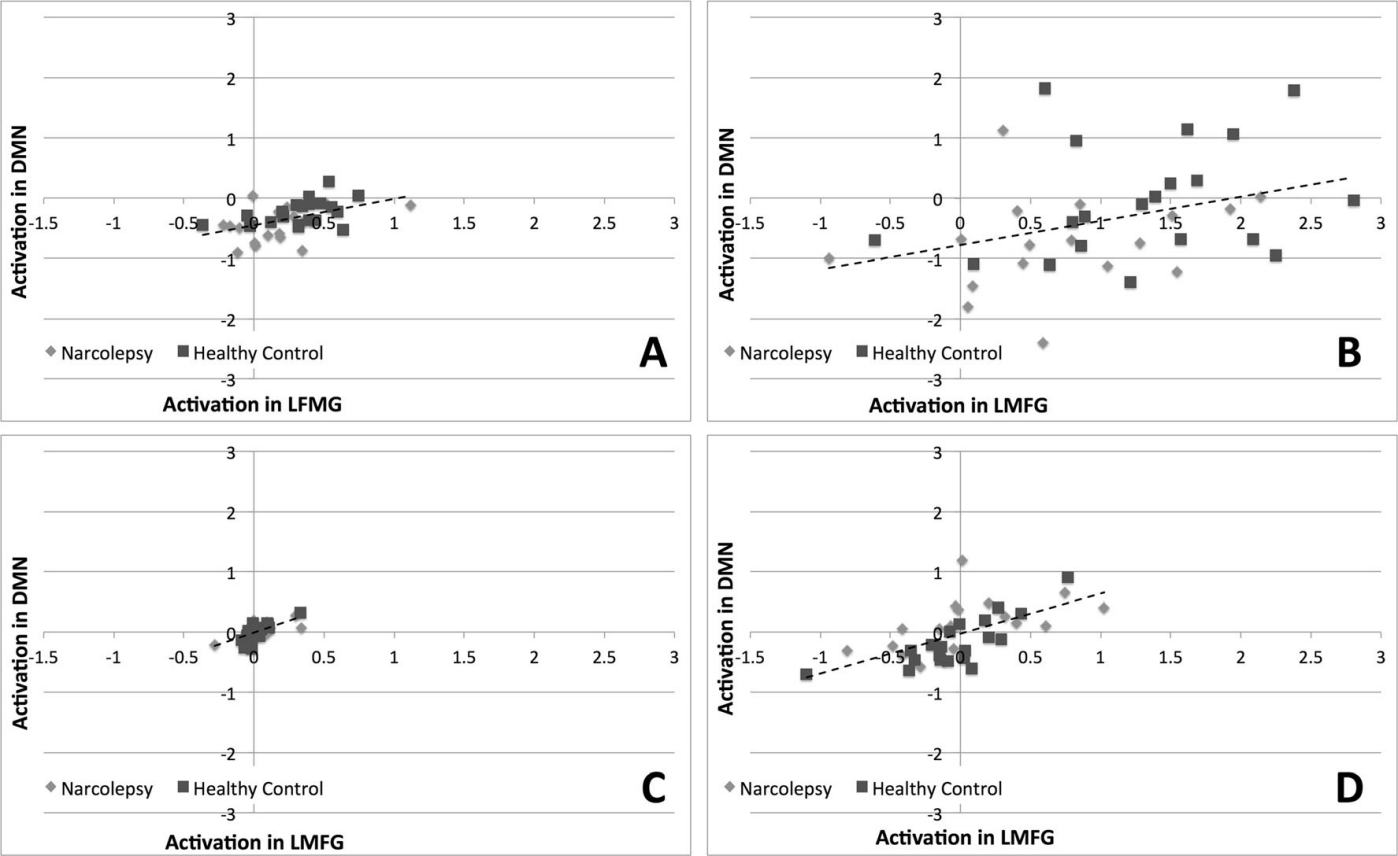
Results from the correlation analysis comparing activation levels in left middle frontal gyrus (LMFG) and DMN. **a** Encoding of sentences. **b** Recognition of words. **c** Parametric effect of load during encoding of sentences. **d** Parametric effect of load during recognition of words. *Dashed lines* indicate best linear fit of all data across both groups. For illustrative purposes, narcolepsy data points are indicated by *light gray diamonds* and healthy controls by *dark gray squares*

**Fig. 4:**
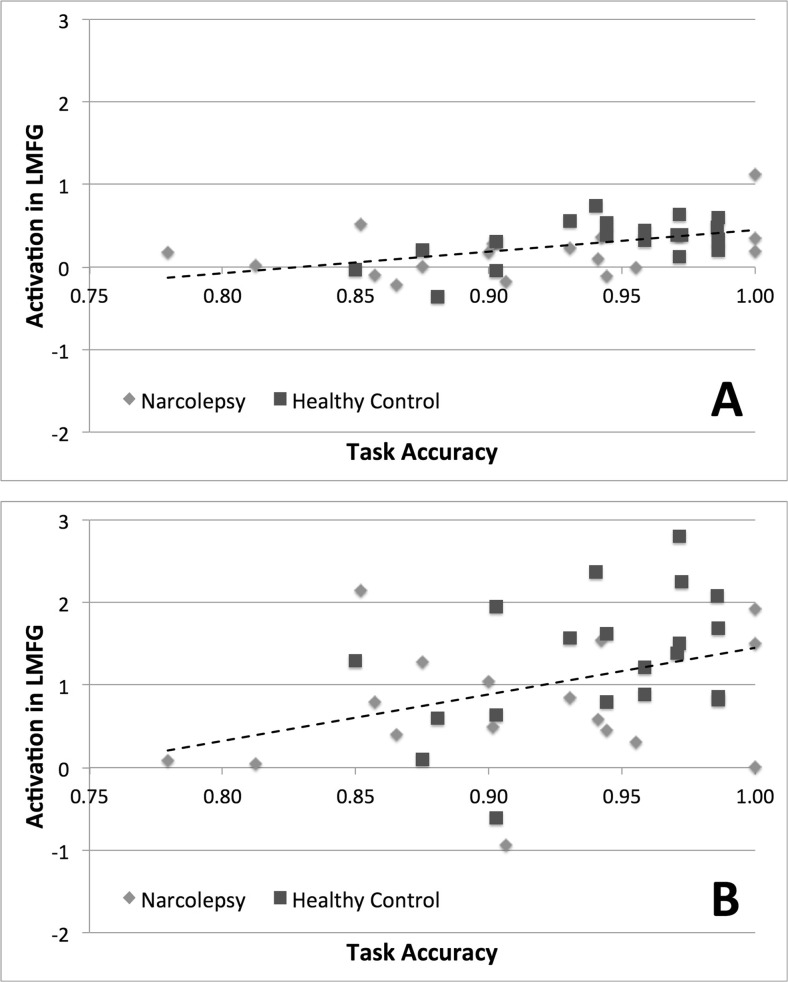
Results from correlation analysis comparing activation levels in LMFG to working memory task accuracy. **a** Encoding of sentences. **b** Recognition of words. *Dashed lines* indicate best linear fit of all data across both groups. For illustrative purposes, narcolepsy data points are indicated by *light gray diamonds* and healthy controls by *dark gray squares*

Results from the correlation analyses (Table 3) revealed that, for all four contrasts of interest, activity within the LMFG ROI was positively correlated with activity in the DMN ROI (Fig. 3a–d). Specifically, activity in the LMFG ROI was significantly correlated with activity in the DMN ROI during the encoding of sentences. The correlation between LMFG and DMN activity was only trend-level during the recognition of words. We also noted for both the encoding of sentences and the recognition of words, activity within the LMFG ROI was positively correlated with overall task accuracy (Fig. 4a–b). Again, this correlation was statistically significant for the encoding of sentences and only trend-level for the recognition of words. No statistically significant or trend-level correlations were noted between task relevant behavior and the DMN ROI.

**Table 3:** Results from the correlation analyses examining the relationship between activation in the LMFG and DMN ROIs and working memory task accuracy. Values are given as Pearson’s *r* coefficients

	DMN	Task accuracy
LMFG: Encoding of sentences	0.481 (*p* < 0.003) **	0.487 (*p* < 0.002) **
LMFG: Recognition of words	0.354 (*p* < 0.032) *	0.37 (*p* < 0.024) *
LMFG: Parametric effect of load, encoding of sentences	0.687 (*p* < 3 × 10^−6^) **	n.s.
LMFG: Parametric effect of load, recognition of words	0.628 (*p* < 3.10 × 10^−5^) **	n.s.

**Significant at Bonferroni correction for comparing across all eight correlations of interest. *Trend-level at *p* < 0.05, uncorrected for multiple comparisons

### Task performance

Results from the between-group repeated measures GLM for the effect of working memory load on overall task accuracy showed no significant effects of load (F_(2,31)_ = 1.96, *n.s*.) nor any significant between-subjects effect of study group (F_(1,32)_ = 0.42, *n.s*.). Estimated marginal means indicated that both groups performed the task with similar levels of accuracy regardless of working memory load (healthy controls: 93.2 ± 0.012%, narcolepsy patients: 92.0 ± 0.013%). No significant study group x load interaction was observed.

Again, for reaction time, no significant effect was observed for working memory load (F_(2,31)_ = 1.18, *n.s*.), however, there was a significant between-subjects effect for study group (F_(1,32)_ = 5.54, *p* < 0.025). Planned comparisons revealed that the healthy controls responded faster than the narcolepsy patients regardless of working memory load (healthy controls: 1110 ± 54 msec, narcolepsy patients: 1308 ± 59 msec). Regression analyses revealed that this difference in reaction time was not due to either the narcolepsy patients progressively getting slower during the task (*t* = 0.55, *n.s.*) or the healthy controls getting faster during the task (*t* = −1.1, *n.s.*). Again, no significant study group x load interaction was observed.

We note that despite the narcolepsy patients having significantly more missed trials than the healthy controls, no significant correlation between the number of missed trials and overall reaction time was observed. There was a trend for the overall task accuracy to be negatively correlated with the number of missed trials, however, when considering the controls and narcolepsy patients separately, this effect appeared to be driven entirely by the healthy controls. No significant relationship was observed between the number of missed trials and overall task accuracy for the narcolepsy patients.

### EEG electrical neuroimaging analysis

**Fig. 5:**
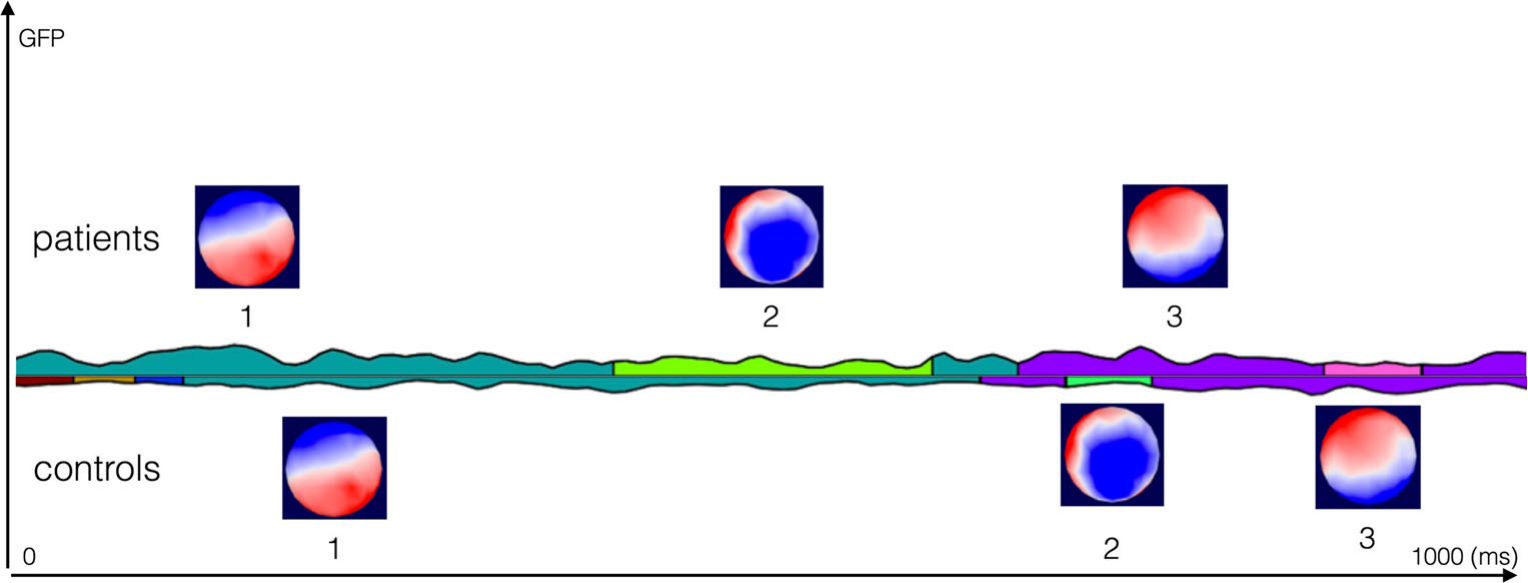
Results from the EEG neuroimaging analysis. The figure represents the results of the microstate segmentation done on the grand means of each group over the 1000 ms EEG epoch, reflecting the total time of the word presentation, where 0 ms indicates the stimulus presentation. The figure shows the three microstates that occurred in both the patient (*top line*) and the control (*bottom line*) group. We did not consider segments with an average duration of less than 10 time frames (80 ms) as physiological, they represent transitional microstates and were also not found to be significant after fitting back on the individual EEG data

The electrical neuroimaging analysis revealed three stable topographies common between both groups (Fig. 5), hereafter referred to as ‘microstates’. In the selected time window, no significant, between-group differences were found for any of the three measures (mean duration, number of occurrences, and global field power).

### GABA+ and glutamate concentrations

**Fig. 6:**
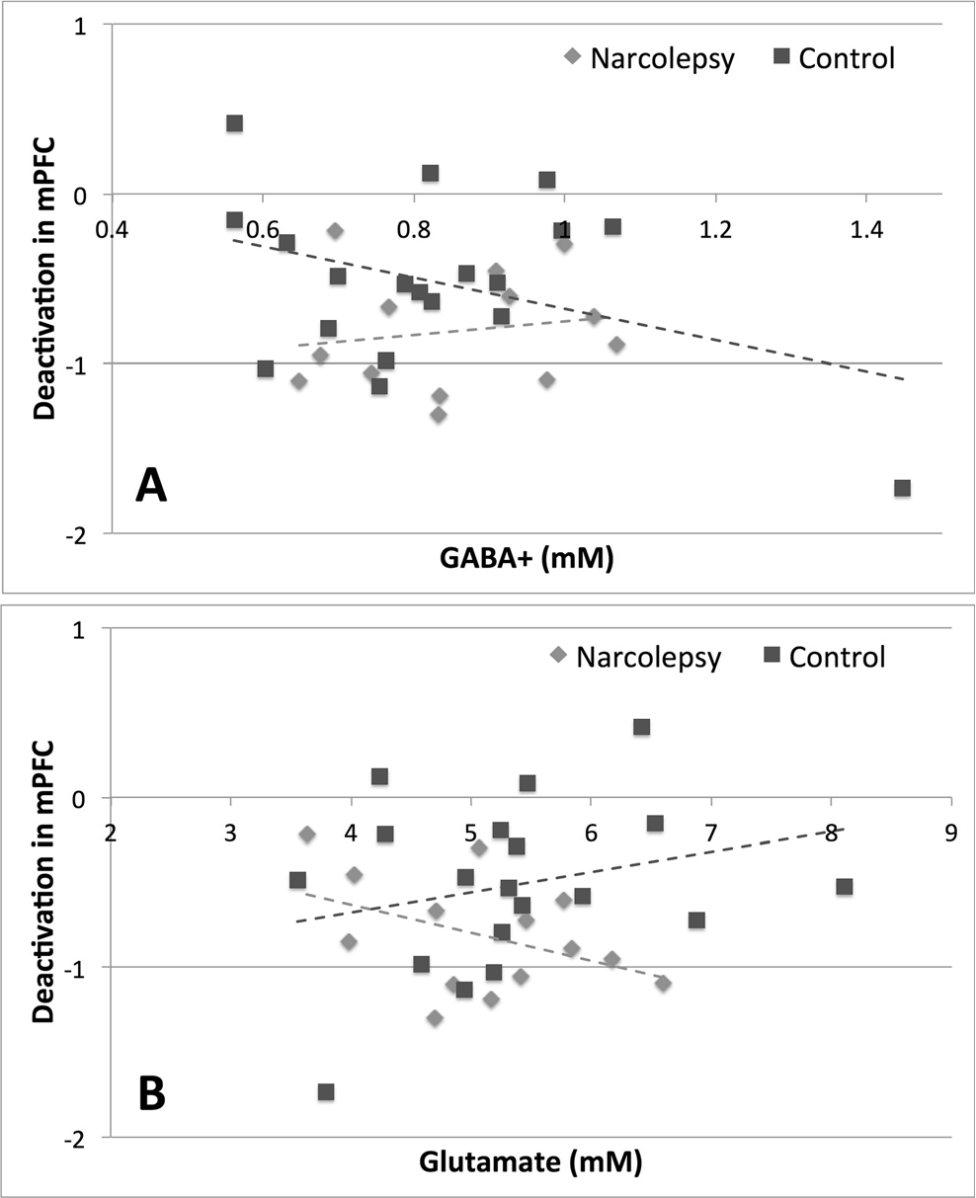
Results from correlation analysis comparing GABA+ and Glutamate concentrations to BOLD activity levels in medial prefrontal cortex during the encoding of sentences. **a** GABA+ with deactivation in medial prefrontal cortex (mPFC). **b** Glutamate with deactivation in mPFC. *Dashed lines* indicate best linear fit, and narcolepsy data points are indicated by *light gray diamonds* and healthy controls by *dark gray squares*

The average GABA+ concentration in the medial prefrontal cortex was found to be 0.85 ± 0.14 mM for the narcolepsy patients and 0.82 ± 0.21 mM for the healthy controls. A two-sample t-test revealed no significant between-group difference (*t* = 0.45, *n.s.*). The average Glutamate concentration was observed to be 5.1 ± 0.86 mM for the patients and 5.3 ± 1.1 mM for the controls. Again no significant between-group difference was noted (*t* = 0.7, *n.s.*). The results from the Pearson correlation analyses are summarized in Table 4. In general, we noted that during the encoding of sentences (Fig. 6), the narcolepsy patients and healthy controls had opposite patterns of correlations for both GABA+ and Glutamate, while for the recognition of words, both groups exhibited the same pattern of correlations for both metabolites. The Fisher’s Z transformation test indicated that there were trend-level, between-group differences (*p* < 0.1) for the correlation coefficients between both GABA+ and Glutamate for the relative level of BOLD deactivation in the medial prefrontal cortex measured during the encoding of sentences. No other between-group differences, trend-level or otherwise, were noted when comparing the Pearson’s correlation coefficients between the patients and controls.

**Table 4:** Results from analysis of the GABA+ and Glutamate MRS data. The table shows both the relative concentrations of each metabolite for each group, as well as, the correlation coefficients between each metabolite concentration and peak areas of task-related activation and deactivation in the medial prefrontal cortex for both the encoding of sentences and the recognition of words

Average metabolite concentrations				
	Narcolepsy patients	Healthy controls	T	p
GABA+	0.85 ± 0.14	0.83 ± 0.21	0.45	n.s
Glutamate	5.1 ± 0.86	5.3 ± 1.1	0.68	n.s
Correlation coefficients between metabolite concentrations and BOLD activation in mPFC				
	Narcolepsy patients	Healthy controls	Z	p
GABA+: mPFC activation, encoding of sentences	0.47	-0.05	1.4	n.s
GABA+: mPFC deactivation, encoding of sentences	0.17	-0.38	1.5	0.1
GABA+: mPFC activation, recognition of words	0.31	0.20	0.3	n.s
GABA+: mPFC deactivation, recognition of words	-0.30	-0.40	0.3	n.s
Glutamate: mPFC activation, encoding of sentences	-0.11	0.29	1.0	n.s
Glutamate: mPFC deactivation, encoding of sentences	-0.42	0.26	1.8	0.07
Glutamate: mPFC activation, recognition of words	0.24	0.30	0.1	n.s
Glutamate: mPFC deactivation, recognition of words	0.14	0.40	0.7	n.s

## Discussion

We present a study examining the neural correlates of cognitive dysfunction in a population of adolescents with narcolepsy to test the primary study hypothesis that decreased deactivation in the DMN would be observed during working memory task performance. Partially confirming this primary study hypothesis, we observed that the narcolepsy patients had altered deactivation within the DMN during performance of a verbal working memory task with varying load compared with age- and gender-matched healthy controls. However, this altered deactivation within the DMN took the form of increased deactivation and was observed in parallel with no measurable deficits in overall task accuracy nor any significant, reduced activity within the LMFG. The main findings of the study can be summarized as follows: 1) patients with narcolepsy were not generally characterized by working memory deficits; 2) patients with narcolepsy were not characterized by altered brain activity in frontal brain regions previously linked to working memory performance; 3) patients with narcolepsy appeared to require increased neuronal resources to perform the working memory task; 4) patients with narcolepsy were characterized by increased deactivation of the DMN, where the level of deactivation in the DMN was positively correlated with activity within task-related frontal regions; and 5) there was trend-level evidence for decreased GABA+ and increased Glutamate levels to correspond with increased deactivation in the medial prefrontal cortex during the encoding of sentences in the narcolepsy patients, while the opposite pattern was observed for healthy controls.

In line with the proposed theory of misbalanced cognitive processing resources but contrary to our primary study hypothesis (Naumann et al. 2006), we observed that cognitive task performance in narcolepsy patients was characterized by increased deactivation of the DMN compared with healthy controls. Typically, task-related increased deactivation of the DMN is linked to increased mental effort (Ceko et al. 2015; Daamen et al. 2015; Newton et al. 2011). This may suggest that either narcolepsy patients find any cognitive task effortful, regardless of actual level of mental effort required, or their cognitive resource allocation system is overly focused on maintaining adequate levels of attention to the detriment of cognitive task performance. Supporting the latter argument for narcolepsy being characterized by a misbalance of cognitive resources, we observed that brain activity within the DMN and LMFG ROIs were significantly positively correlated with each other during the encoding of sentences, indicating that increased deactivation within the DMN was associated with decreased activity within the left middle frontal gyrus for the aspect of the working memory task requiring sustained attention. For the recognition of words, this correlation was only trend-level. However, the general pattern was for activity with the DMN and LMFG to be positively correlated during the working memory task as a whole. Additionally, we noted that while in healthy controls increased levels of deactivation during the encoding of sentences correlated with increased concentrations of GABA+ (as is typically observed e.g., (Hu et al. 2013; Northoff et al. 2007) and decreased concentrations of Glutamate, the opposite was observed for the narcolepsy patients. In the patients, it appeared as if increased levels of deactivation in the medial prefrontal cortex during the encoding of sentences was related to decreased concentrations of GABA+ and increased concentrations of Glutamate, again pointing towards an active suppression or some form of metabolic dysregulation of at least the anterior portion of the default mode network. We should be careful to note that the between-group differences in the respective relationships between GABA+ and Glutamate and deactivation in the medial ACC during task performance were only trend-level. However, given the evidence pointing towards a dysfunction in the DMN from the task-based fMRI data, particularly with regards to sustained attention, these MRS results cannot wholly be dismissed solely on grounds of lack of statistical significance. Taken together, the fMRI and MRS results suggest a misbalance in the allocation of cognitive resources during sustained attention related to the DMN, pointing to a potential target of therapy, as a handful of studies have shown that DMN activity levels can be actively manipulated improving cognitive performance (Zhang et al. 2015a, b).

In the situation where one would expect increased deactivation in the DMN to be related to increased working memory load, we instead observed that while the healthy controls generally followed the previously published pattern of increased deactivation of the DMN with increased working memory load during the recognition of words, the narcolepsy patients exhibited increased activation within the default mode network as working memory load requirements increased. A number of studies have begun to suggest that activity within the default mode network can facilitate working memory performance (e.g., (Piccoli et al. 2015), so it is unclear whether the currently observed increased activity within the task negative network in narcolepsy patients is a result of them making use of the default mode network to perform the task or the release of the resources being used to otherwise suppress default mode network activity in favor of allocation towards task positive related brain regions. At the very least, this finding adds evidence supporting the notion that narcolepsy is characterized by deficits in the adequate sharing of cognitive resources between task performance and attention monitoring.

In line with previously published studies on working memory ability in narcolepsy patients (Henry et al. 1993; Naumann et al. 2006), our results from the standard cognitive test battery administered prior to MRI scanning and the working memory task performed during the fMRI scan showed that the narcolepsy patients performed all tasks with similar accuracy as the healthy controls. The only measurable difference in fMRI working memory task performance was observed as increased reaction times on the part of the narcolepsy patients. At least one study (Henry et al. 1993) has interpreted increased response times during performance of a working memory task as slowing of information processing, not otherwise related to motor slowing, tiredness, or a global reduction in arousal. While our study was not specifically designed to investigate processing speed, our results seem to point in this direction, as we observed increased reaction time for the narcolepsy patients that appeared to be independent of working memory load, task duration, or number of missed trials. Other than the significantly increased likelihood of the narcolepsy patients to fall asleep during the fMRI scans (or at least to stop participating in the task), our behavioral results also did not suggest any particular deficit in the ability to sustain attention at a high level over moderate lengths of time, as has been previously reported (Naumann and Daum 2003).

The lack of any observed decreased activity within the LMFG during working memory task performance also supports the view that narcolepsy is generally not characterized by any deficits in cognition beyond those previously observed for sustained attention. It is unclear whether our negative findings are in line with results from previous studies examining working memory function in narcolepsy, as the two published to date have been essentially case studies and focused on specific aspects of task performance manipulation, specifically mental fatigue (Thomas 2005) and normative effects of stimulant medication (Allen et al. 2012). We do note, though, that the current results stand in contrast to our previously published results with this same task finding a working memory deficit in patients with periodic idiopathic hypersomia (Kleine Levin Syndrome), another sleep disorder with typical onset in adolescence (Engstrom et al. 2009, 2013). It is well documented that working memory is subserved by a core set of brain regions (Rottschy et al. 2012) – with considerable historical emphasis placed on the left dorsolateral prefrontal cortex as being one of the key working memory brain regions (D'Esposito et al. 1995) – so, it may be our negative results are due to our decision to solely focus on this region. However, given that brain activity within our chosen LMFG ROI was significantly correlated with task accuracy during the encoding aspects of our task (and had a trend-level correlation with the retrieval aspect of the task), we were confident that any altered working memory-related brain activity would have been apparent in this region. Instead as described above, abnormalities in brain activation patterns during cognitive task performance indicated a misbalance of cognitive resources towards maintaining and monitoring sustained attention.

There are several limitations to the current study, most notably the somewhat small sample size preventing either between-group analyses corrected at the whole brain level or more extensive region-of-interest analyses. However, given that the previous two fMRI studies examining cognitive function in narcolepsy patients included one or two patients and no control groups, we feel that the current study presents a significant step forward towards a more complete understanding of cognitive deficits in narcolepsy. We also note that the narcolepsy sample was mixed in terms of the presence of depression and depressive symptoms at the time of scanning. Major depressive disorder is known to both affect cognitive performance and alter the associated brain function (Lee et al. 2012; Wang et al. 2015). Finally, we allowed the narcolepsy patients to take their prescribed medication during the study. Studies examining the effects of the most commonly prescribed stimulant medication (modafinil) in patients with narcolepsy present mixed findings, with some observing positive effects (Becker et al. 2004) and others no effects (Muller et al. 2004). However, it must be noted, this decision to allow medication use may have had an impact on the presented results. Future studies should, in a larger population, look to study both the effects of psychiatric comorbidities and medication status on cognitive function in narcolepsy.

## Conclusions

We have presented evidence that supports the theory that cognitive dysfunction in narcolepsy stems from a misbalance or misallocation of cognitive resources in favor of maintaining and monitoring sustained attention levels. Specifically, we observed that the narcoleptic patients had significantly increased deactivation within the default mode network, even with intact task performance accuracy. Additionally, we note that data from GABA-MRS appear to support this notion that the increased deactivation within the default mode network may be the result of an active process. We did not observe any evidence for a true working memory deficit in narcolepsy in the functional neuroimaging data, indicating that the source of self-reported cognitive difficulties may stem from a dysregulation in the sustained attention system.

## Electronic supplementary material

Supplemental Table 1 (DOCX 82 kb)
