# Supplementary material for: Evidence for cognitive resource imbalance in adolescents with narcolepsy
Source: Brain Imaging Behav. 2017 Mar 20;12(2):411–24. doi: 10.1007/s11682-017-9706-y (PMC5880867; doi:10.1007/s11682-017-9706-y)
Supplement: Supplementary file 1 — (DOCX 82 kb) [file 11682_2017_9706_MOESM1_ESM.docx]

Supplemental Table 1. Stereotactic peak coordinates for the four task regressors of interest from the verbal working memory tasks. A. Encoding of Sentences. B. Recognition of Words. C. Parametric effect of load on sentence encoding. D. Parametric effect of load on word recognition.

**A. Encoding of Sentences**

| Region | BA | X | Y | Z | T |
| --- | --- | --- | --- | --- | --- |
| Activations | | | | | |
| Medial Frontal Gyrus/SMA | 8 | 2 | 22 | 46 | 10.25 |
| L IFG | 9 | -40 | 8 | 26 | 13.27 |
|  | 45 | -56 | 22 | 6 | 13.23 |
| R IFG/MFG |  | 50 | 20 | 26 | 6.46 |
| L Insula |  | -32 | 22 | -8 | 11.20 |
| R Insula |  | 34 | 22 | -8 | 9.76 |
| L Lingual Gyrus/Calcarine/Cuneus | 17 | -10 | -96 | -4 | 13.99 |
| R Calcarine Fissure/Cuneus | 17 | 22 | -100 | -2 | 18.32 |
| L Hippocampus |  | -24 | -30 | -4 | 11.81 |
| R Hippocampus |  | 24 | -26 | -6 | 6.70 |
| L Thalamus |  | -12 | -14 | 8 | 9.53 |
| R Thalamus |  | 12 | -10 | 8 | 5.69 |
|  |  |  |  |  |  |
| Deactivations | | | | | |
| ACC | 10 | 6 | 54 | -4 | 8.18 |
| Cingulate Gyrus/Precuneus | 31 | 6 | -24 | 46 | 12.16 |
| L STG |  | -44 | -14 | -4 | 11.28 |
| R STG |  | 42 | -12 | -6 | 8.30 |
|  |  |  |  |  |  |

**B. Recognition of Words**

| Region | BA | X | Y | Z | T |
| --- | --- | --- | --- | --- | --- |
| Activations | | | | | |
| Medial Frontal Gyrus/SMA/ACC | 8, 32 | 0 | 28 | 46 | 14.01 |
| L MFG/IFG | 9 | -42 | 24 | 22 | 7.49 |
| R MFG | 9 | 48 | 22 | 34 | 7.98 |
| L IFG/Insula |  | -32 | 20 | -6 | 11.44 |
| R IFG/Insula | 47 | 36 | 22 | -8 | 10.73 |
| L IPL | 40 | -40 | -48 | 44 | 10.41 |
| R IPL | 40 | 50 | -44 | 46 | 9.65 |
| L Thalamus |  | -14 | -18 | 6 | 7.81 |
| R Thalamus |  | 14 | -8 | 6 | 5.92 |
|  |  |  |  |  |  |
| Deactivations | | | | | |
| Medial Frontal Gyrus/ACC | 10 | 0 | 52 | -4 | 7.20 |
| R Posterior Insula | 13 | 42 | -14 | -6 | 6.46 |
| Posterior Cingulate Cortex/Precuneus | 31 | -6 | -60 | 22 | 5.81 |
| L Lingual Gyrus |  | -12 | -92 | -8 | 9.02 |
| R Lingual Gyrus |  | 16 | -90 | -4 | 8.80 |
|  |  |  |  |  |  |

**C. Parametric effect of load on sentence encoding**

| Region | BA | X | Y | Z | T |
| --- | --- | --- | --- | --- | --- |
| L Lingual Gyrus |  | -8 | -94 | 2 | 7.27 |
| R Calcarine Fissure/Cuneus |  | 10 | -94 | 2 | 5.79 |
| L MTG/STG | 22 | -60 | -34 | 6 | 7.02 |

**D. Parametric effect of load on word recognition**

| Region | BA | X | Y | Z | T |
| --- | --- | --- | --- | --- | --- |
| R Fusiform Gyrus |  | 3 | -60 | -14 | 6.19 |
